# Rumen by-pass soybean meal reduced ruminal ammonia but did not improve growth performance and nitrogen utilization in growing Hanwoo heifers

**DOI:** 10.5713/ab.24.0860

**Published:** 2025-04-11

**Authors:** Joonpyo Oh, Dae Kyum Yoo, Si Woo Jeong, Hyun Wook Shin, Jong Min Kim, Ja-Kyeom Seo

**Affiliations:** 1 Cargill Animal Nutrition Korea, Seongnam, Korea; 2 Department of Animal Science, Life and Industry Convergence Research Institute, Pusan National University, Miryang, Korea

**Keywords:** Growth Performance, Hanwoo Heifer, Nitrogen Utilization, Protein Protection, Rumen Fermentation

## Abstract

**Objective:**

The effects of heat-treated soybean meal (SBM) and citric acid (HCSBM) on the growth performance, rumen fermentation, blood metabolites, nutrient digestibility, and nitrogen (N) utilization were evaluated in growing Hanwoo heifers.

**Methods:**

Eight growing Hanwoo heifers (initial body weight: 228.5±11.3 kg; age: 9.3±0.3 months) were allocated to a crossover design with two dietary treatments: a control diet containing untreated SBM (Control) and a diet containing HCSBM. There were two 28-day phases in the trial, each containing 14-day measurements and an adaptation period. Growth performance, rumen fermentation parameters, blood metabolites, hematological parameters, apparent digestibility, energy, and N utilization were measured.

**Results:**

No significant differences were observed in the growth performance or energy utilization between the diets. The digestibility of dry matter and crude protein (CP) did not differ between the two diets. HCSBM supplementation increased neutral detergent fiber digestibility (p = 0.0874) and acetate molar proportions (p = 0.0748). The HCSBM diet resulted in lower ruminal ammonia nitrogen concentrations and iso-valerate proportions (p<0.05), indicating reduced ruminal protein degradation. Blood metabolites related to protein metabolism showed no significant differences between treatments. The control group exhibited higher red blood cell counts and hemoglobin and hematocrit levels (p<0.05). N excretion or retention did not significantly differ between the dietary groups.

**Conclusion:**

Despite enhanced protection against ruminal protein degradation, HCSBM supplementation did not improve N utilization efficiency or growth performance in growing Hanwoo heifers, possibly because of a sufficient N supply from the high CP content in both diets.

## INTRODUCTION

Over the past decade, the Hanwoo cattle population in South Korea has steadily increased, raising environmental concerns regarding ruminant production. Nitrogen (N) is an essential nutrient for animal growth and productivity [1]. However, ruminants have lower N utilization efficiency than do other farm animals because of inefficient use of N in the rumen [1]. Consequently, a large portion of dietary N is excreted, contributing to environmental concerns such as greenhouse gas production, and the eutrophication of water and soil [2]. These effects are particularly evident when cattle are fed diets rich in rumen-degrading proteins.

Soybean meal (SBM) serves as the primary protein source in the feed of both beef and dairy cattle and is valued for its balanced amino acid composition. However, 60–70% of the crude protein (CP) in SBM is degraded in the rumen [3], therefore strategies to protect SBM from rumen degradation are required. Recent studies have explored methods to reduce ruminal degradation of SBM by combining physical and chemical treatments. Combining heat treatment with xylose, malic acid, and orthophosphoric acid reduces rumen protein degradation in protein feeds including peanut meal, sunflower seeds, and sunflower meal [4,5]. However, the combination of malic acid and heat treatment applied to SBM did not protect against rumen degradation [3].

We previously evaluated the rumen protective effects of heat and citric acid (CA) treatment on SBM using *in vitro* methods. The results indicated that SBM treated with heat and CA (HCSBM) led to significantly lower ruminal CP degradability and ammonia nitrogen (NH_3_-N) concentrations than in untreated SBM [2]. To validate these findings and assess their practical applications in ruminant nutrition, we examined growing Hanwoo heifers, which are at a critical stage of musculoskeletal development and require an adequate protein supply [6]. Therefore, we assessed the effect of diets supplemented with rumen bypass SBM on growth performance, rumen fermentation, blood metabolites, digestibility, and N utilization in growing Hanwoo heifers.

## MATERIALS AND METHODS

Protocols for animal use in this study were reviewed and approved by the Animal Research Ethics Committee of Pusan National University (PNU-2024-0454).

### Formulation and chemical analysis of experimental diets

The main components of the two experimental diets were a commercial concentrate mix (Famsco Co., Ltd., Chilgok, Korea), oat hay, SBM, and HCSBM. The chemical composition of the commercial concentrate mix, expressed on a dry matter (DM) basis, was as follows: CP 17.5%, neutral detergent fiber (NDF) 33.2%, acid detergent fiber (ADF) 16.1%, lignin 6.95%, and ether extract (EE) 4.82%. HCSBM was produced by adding 400 mL of 1.5 mol/L CA solution per kg of SBM, followed by heat treatment at 220°C for 1 h in a roaster (FEC-006; Biotech, Gimpo, Korea). Details of the diet formulations and their chemical compositions are presented in Table 1. Both experimental diets were designed to meet or exceed the target average daily gain (ADG) of 0.8 kg/d, following National Academies of Sciences, Engineering, and Medicine (NASEM) [7] guidelines. All feed samples and experimental diets were ground using a cyclone mill (Foss Tecator Cyclotec 1093; Foss, Hillerød, Denmark) fitted with a 1 mm screen before chemical analysis and dried at 65°C for 72 h. Ash (#942.05), EE (#920.39), and CP (#990.03) were analyzed according to the procedures outlined by the Association of Official Analytical Chemists International [8]. The N content was determined using the Kjeldahl method. The ADF, lignin, and NDF were analyzed as described by Van Soest et al [9].

### Design of *in vivo* experiments

**Figure 1:**
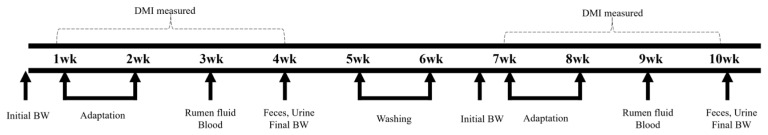
Brief schedule of *in vivo* experiment. DMI, dry matter intake; BW, body weight.

The study employed a crossover design with eight growing Hanwoo heifers (initial body weight (BW): 228.5±11.3 kg; age: 9.3±0.3 months). The heifers were randomly allocated to two groups of four, with one heifer per pen. The experiment consisted of two 28 d periods, during which different dietary treatments were administered. A 14 d washout period was implemented between the two treatment phases to minimize carryover effects. Each 28 d period was divided into an adaptation phase (1 to 14 d), during which the heifers were acclimated to the diets, and a measurement phase (15 to 28 d), during which data were collected (Figure 1). Water and mineral blocks were provided *ad libitum*. The experimental diets were offered to the heifers twice daily at 08:00 and 16:00. Feed intake was managed to allow *ad libitum* consumption to achieve approximately 10% feed refusal. Daily dry matter intake (DMI) was calculated by subtracting the amount of refused feed from the total amount provided in each pen. The BWs of all Hanwoo heifers were measured before feeding on the first morning of each period, and final weights were measured on the last day of each period. These measurements were used to calculate the feed conversion rate (FCR) and ADG.

### Rumen fermentation characteristics

Rumen fluid samples were collected using an oral stomach tube at 8 h intervals, starting 2 h before morning feeding, between day 16 and 18 of each period. The rumen fluid pH was measured using a portable pH meter (ST300; Ohaus, Parsippany, NJ, USA), and the samples were promptly transported to the laboratory. Rumen fluid was centrifuged at 3,500 rpm for 20 min at 4°C. The supernatant was divided into aliquots to assess volatile fatty acids (VFA) and NH_3_-N. VFA and NH_3_-N were analyzed using pooled ruminal fluid. Preprocessing and analytical methods for VFA and NH_3_-N were performed as described by Yoo et al [10].

### Blood metabolites and hematological parameters

Blood samples were collected from the jugular vein in the morning, before feeding. The samples were aliquoted into serum tubes (BD Vacutainer; BD Biosciences, Franklin Lakes, NJ, USA) containing a clot activator and into microtubes (Mini Collect Tube 0.25/0.5 mL K_3_EDTA; Greiner, Kremsmünster, Austria) containing EDTA. The blood samples were centrifuged in serum tubes at 2,500×g for 15 min at 4°C. Serum parameters including total protein, blood urea nitrogen (BUN), creatinine (Crea), albumin (Alb), inorganic phosphate (IP), triglycerides, alanine aminotransferase, calcium (Ca), magnesium (Mg), glucose, and aspartate aminotransferase were analyzed using an automated biochemistry analyzer (TBA-40FR, Toshiba Instruments, Tokyo, Japan). Additionally, each blood sample was assessed for white blood cells (WBC), basophils, neutrophils, lymphocytes, monocytes, eosinophils, red blood cells (RBC), hemoglobin (HGB), hematocrit (HCT), and platelets using an automated hematology analyzer (VetScan HM5; Abaxis, Inc., Union City, CA, USA).

### Apparent digestibility and energy utilization

Fecal spot samples (minimum 200 g) were collected at 3 h intervals (08:00, 11:00, 14:00, 17:00, 20:00, 23:00, 02:00, and 05:00 h) relative to the morning feeding time. Feed samples were collected during the study period. Both the feed and fecal samples were dried at 60°C for 72 h. After drying, the fecal samples were pooled, and both the feed and pooled fecal samples were ground using a cyclone mill with a 1 mm screen. Indigestible NDF (iNDF) was used as a marker to estimate apparent digestibility. To determine iNDF, the feed and fecal samples (0.5 gDM) were placed in F57 bags (25 μm pore size; Ankom Technology Corp., Macedon, NY, USA) and incubated in the rumen of two cannulated Holstein steers (BW: 650±12.3 kg) for 12 d. The steers were fed a diet consisting of 60:40 commercial concentrate (Famsco Co., Ltd.) and oat hay, provided twice daily with *ad libitum* access to water and mineral blocks. After incubation, the bags were thoroughly rinsed until the water was clear. The NDF, CP, and N contents of the feed and fecal samples were analyzed using previously described methods. The energy content of the feed and fecal samples was assessed using a Parr 6400 Automatic Isoperibol Calorimeter (Parr Instrument Co., Moline, IL, USA), according to the manufacturer’s guidelines, to determine both gross energy (GE) and digestible energy (DE). The apparent digestibility and DE were calculated using the equations described by Beck et al [11].

### Estimation of urine output and nitrogen retention

Urine spot sampling was conducted at the same intervals as fecal spot sampling, and the urine samples were filtered through four layers of cheesecloth. Urine samples (minimum of 200 mL) were mixed (pH <3) with 2 mL of 2 M H_2_SO_4_ and 38 mL of urine for N analysis. In addition, 40 mL of urine was aliquoted for Crea analysis. All samples were stored at −20°C until analysis. Urine samples collected at each time point were pooled and analyzed for N content using the Kjeldahl method. Urine Crea concentrations at each time point were determined using an automated biochemical analyzer. Urine output was estimated using Crea concentration as a marker, and calculations were performed as described by Barbosa et al [12].


$$\text{Urine output }(\text{kg}/\text{d})=27.11\times {\text{BW}}^{0.75}÷[\text{creatinine concentration }(\text{mg}/\text{dL})\times 10]$$

N retention was estimated using the following equation:


N retention (% of N intake)=[N intake (g/d)-(urine N (g/d)+fecal N(g/d))÷N intake(g/d)]×100

### Statistical analysis

The Shapiro–Wilk test in SAS version 9.4 (SAS Institute Inc., Cary, NC, USA) was used to assess the normality of all parameters, which were confirmed to follow a normal distribution. The PROC MIXED procedure in SAS 9.4 (SAS Institute, Cary, NC, USA) was used for all data analyses. The model used was:


$$Y_{ijk}=\mu +T_{i}+P_{j}+A_{k}+{ɛ}_{ijk},$$

where *Y_ijk_* is the response variable, *μ* is the overall mean, *T_i_* is the fixed effect of treatment (*i* = CON vs. HCSBM), *P_j_* is the random effect of period (*j* = 1, 2), *A_k_* is the random effect of animal (*k* = 1–8), and ε*_ijk_* is the residual error. Pairwise comparisons of least squares means were performed using the PDIFF option with Tukey-Kramer adjustment. The significance level was set at p<0.05, with trends considered when 0.05 ≤ p<0.1.

## RESULTS AND DISCUSSION

Heat treatment of protein sources triggers Maillard reactions, leading to an increase in the ruminal undegradable protein content [13]. Similarly, it has been suggested that acid treatment induces structural changes in proteins, thereby providing enhanced protection against ruminal degradation [14]. When these treatments were combined, the protective effect against ruminal protein degradation might be significantly greater than that of either treatment alone. Our previous *in vitro* study confirmed that combined treatment of HCSBM significantly reduces CP degradation and NH_3_-N concentrations in the rumen [2]. Based on these findings, we hypothesized that feeding HCSBM produced through heat and CA treatment would protect proteins from degradation in the rumen, potentially increasing N availability and growth performance.

The results of growth performance, apparent nutrient digestibility, and energy utilization are shown in Table 2. There were no significant differences between the two treatment groups for total DMI, concentrate DMI, forage DMI, ADG, and FCR. Although DM digestibility, CP digestibility, GE intake, and DE were similar between the Control and HCSBM groups, NDF digestibility tended to be higher in the HCSBM (p = 0.0874). Kazemi-Bonchenari et al [15] reported that supplementing a high-grain diet with approximately 143 g CA per day improved weight gain and feed efficiency in male Holstein calves. Similarly, Wang et al [16] suggested that providing Chinese Simmental steers 200 g of CA per day increased nutrient digestibility. Unlike these studies, we found that daily supplementation with approximately 81 g CA, as the mixed form with SBM, had no positive effects on DM and CP digestibility, ADG, or FCR. This discrepancy may be attributed to the lower CA levels used in the present study. Previous studies demonstrated that addition of CA to the diet improves NDF digestibility [15,16]. Consistent with previous studies, we also showed a tendency toward higher NDF digestibility in HCSBM supplemented with CA. Yoo et al [2] observed that the abundance of the fiber-degrading bacteria *Fibrobacter* tended to be higher in HCSBM than in SBM. Overall, the HCSBM diet tended to show higher fiber digestibility than the Control diet, but DMI, nutrient digestibility, GE intake, and DE were similar. Therefore, it is presumed that feeding HCSBM does not have a positive effect on ADG and FCR.

The ruminal fermentation parameters are presented in Table 3. The NH_3_-N concentration in the rumen is a byproduct of microbial protein utilization and serves as an indicator of protein degradation. Yoo et al [2] found through *in vitro* experiments that ruminal NH_3_-N concentrations were lower in HCSBM than in SBM. Consistent with previous studies, we found that NH_3_-N concentrations were significantly lower in the HCSBM-fed group than in the Control group (p<0.01). Branched-chain VFAs such as isobutyrate and isovalerate are commonly used as indicators of ruminal protein degradation [17]. *In vitro*, the molar proportions of isobutyrate and isovalerate were lower in HCSBM than in SBM [2]. In our study, the molar proportion of isobutyrate did not differ between diets, but the isovalerate proportion was lower in the HCSBM diet than in the Control diet (p<0.05). These findings suggest that the diet containing HCSBM had lower ruminal protein degradability than those in basal diet. Based on the results of DMI and nutrient digestibility results, we predicted no difference in the total VFA production between the two dietary groups, and our findings were consistent with the expected outcomes. The molar proportion of acetate tended to be higher in the HCSBM-dietary group than in the Control diet group (p = 0.075), although no differences were observed in the molar proportions of propionate and butyrate. The higher proportion of acetate in the HCSBM diet than in the Control diet may be attributed to two factors. First, ruminal microbes likely rapidly convert CA to CO_2_ and acetate [18], thereby influencing acetate production. Second, fiber is primarily degraded by fiber-degrading bacteria, with acetate as the predominant fermentation product [19]. The observed tendency for higher NDF digestibility in the HCSBM group likely contributed to the increased molar proportion of acetate.

The results of blood metabolite analysis are shown in Table 4. Ca, IP, and Mg are essential minerals with critical roles in digestion and skeletal development [20]. Mg levels tended to be higher in the Control diet than in the HCSBM diet (p = 0.077), whereas no significant differences were observed between the Control and HCSBM diets for Ca and IP. Burr and Thomas [21] found that addition of 1% CA to the diet of Ayrshire heifer calves reduced their serum Mg concentrations. The reduced Mg levels in the HCSBM diet may be attributed to the effects of CA. The ranges previously reported for Ca (8.0 to 9.7 mg/dL), IP (7.0 to 8.70 mg/dL), and Mg (2.0 to 2.1 mg/dL) during the growth period of Hanwoo cattle are comparable to our findings [22,23]. Previous studies suggested that BUN and Alb represent the relative ratios of protein deposition in the carcass [24] and that Crea concentrations are positively associated with the amount of muscle in carcass accumulation [25]. Our study revealed no significant differences in the BUN, Alb, or Crea levels between the two dietary groups. Therefore, supplementation of HCSBM may not positively affect the N utilization efficiency.

The analysis results of hematological parameters are shown in Table 5. The leukocyte profile showed no significant differences between the two dietary groups. WBC are essential components of the immune system. Previous studies reported a WBC count range of 8.57 to 11.4 (10^9^/L) in the growth phase (approximately 13 months) of Hanwoo heifers [26,27]. We observed lower WBC counts in both dietary groups than those reported in previous studies (Control, 6.16×10^9^/L vs HCSBM, 5.48×10^9^/L). Hematological values in livestock are influenced by factors such as age, climate, feeding management, environmental conditions, and the presence of infections [28]. Considering the normal growth performance and absence of clinical symptoms in both feeding groups, the lower WBC counts observed in this study may not indicate any health concerns. The Control diet led to a significant increase in RBC counts compared to the HCSBM diet (Control, 8.90×10^12^/L vs HCSBM, 8.21×10^12^/L; p<0.05), leading to higher HGB and HCT levels in the animals on the Control diet (p<0.05). RBC, HGB, and HCT are key parameters for assessing the oxygen transport capacity and diagnosing anemia in livestock [28]. Previous studies documented RBC counts between 8.0 and 9.1 (10^12^/L) in growing Hanwoo heifers [26,27]. The reason for the higher RBC counts in the Control diet than in the HCSBM diet is not clearly understood; however, the values from both dietary groups were consistent with previously reported reference ranges.

The estimated N excretion and utilization results are listed in Table 6. Although urinary Crea obtained from spot urine sampling may not accurately estimate the total urine volume as a marker, it enables comparisons across dietary treatments [29]. The estimated urine output tended to be higher in the HCSBM diet than in the Control diet (p = 0.099). Bannik et al [30] suggested that the mineral load that must be excreted, particularly Na and K, as well as N and water intake, significantly affects urine output in cattle. N intake did not differ between the two diets, and mineral blocks and water were provided *ad libitum*. Thus, the tendency for increased urine output in the HCSBM diet group may have been influenced by mineral and water intake; however, as intake levels were not measured, further research is needed to confirm this potential effect. Based on the lower NH_3_-N concentration and molar proportion of isovalerate, the HCSBM diet demonstrated enhanced protein protection from ruminal degradation compared to the Control diet. These findings suggest that improved protein bypass into the small intestine can lead to enhanced protein absorption and utilization efficiency. Given these observations, we predicted that N excretion through urine and feces would decrease, resulting in increased N retention. However, there were no significant differences between the groups consuming the two diets in terms of estimated urinary N, fecal N, and N retention. Therefore, considering the results of N excretion, N retention, BUN, Alb, and Crea, despite enhanced ruminal protein protection, the HCSBM diet may not effectively improve protein absorption and utilization efficiency in the small intestine. According to previous studies, the high CP content range in the diet of growing Hanwoo cattle was 15.0–15.7% [6,26], which was comparable to the CP levels in our study. The absence of differences between the HCSBM and Control diets may be attributed to the sufficient N supply given the high CP content in both diets. Further research utilizing various CP levels is necessary to determine their potential effects on the observed parameters.

## CONCLUSION

Despite enhanced protection against ruminal protein degradation, the HCSBM diet did not appear to improve protein absorption and utilization efficiency in the small intestine, as indicated by the growth performance, BUN, Alb, Crea, N excretion, and N retention results. This lack of improvement may be attributed to a sufficient N supply due to the high CP content in both experimental diets. Further studies of varying CP levels are necessary to clarify the potential effects of HCSBM supplementation on these parameters.

## Figures and Tables

**Table 1:** Diet formulation and chemical composition of the experimental diets

Items	Treatments^1)^	

	Control	HCSBM
Ingredients (% as fed)		
Commercial concentrate^2)^	60.0	60.0
Oat hay	30.0	30.0
Soybean meal	10.0	0
Heat-citric acid-treated soybean meal	0	10.0
Chemical composition		
DM (%)	89.4	90.3
CP (%DM)	16.4	16.1
NDF (%DM)	36.2	36.4
ADF (%DM)	21.0	21.3
Lignin (%DM)	4.97	4.98
EE (%DM)	3.55	3.45
Ash (%DM)	6.35	6.15
TDN (%DM)	70.1	70.0
NEm (Mcal/kg of DM)^3)^	1.68	1.68

1) Control, feed containing 10% soybean meal; HCSBM, feed containing 10% heat-citric acid-treated soybean meal.

2) Commercial concentration was purchased from a feed company (Famsco, Co., Ltd., Chilgok, Korea).

3) Calculated by using the equation from NASEM [7].

DM, dry matter; CP, crude protein; NDF, neutral detergent fiber analyzed with heat stable α-amylase; ADF, acid detergent fiber; EE, ether extract; TDN, total digestible nutrients; NEm, net energy for maintenance.

**Table 2:** Growth performance, apparent digestibility of nutrients, and energy utilization in growing Hanwoo heifers fed experimental diets

Items	Treatments^1)^		SEM	p-value^2)^

	Control	HCSBM		
Growth performance				
Initial BW (kg)	247	249	3.9	0.734
Final BW (kg)	276	278	4.1	0.704
TDMI (kg/d)	7.26	7.06	0.359	0.582
CDMI (kg/d)	5.13	5.00	0.230	0.593
FDMI (kg/d)	2.13	2.06	0.132	0.592
ADG (g/d)	1033	1042	54.1	0.875
FCR	7.15	6.85	0.515	0.577
Apparent digestibility				
DM (%)	70.4	72.0	1.41	0.311
CP (%)	75.8	74.6	0.99	0.272
NDF (%)	53.9	59.0	2.49	0.087
Energy utilization				
GE intake (Mcal/d)	29.1	29.4	1.48	0.863
DE (%)	70.8	72.2	1.33	0.336

1) Control, feed containing 10% soybean meal; HCSBM, feed containing 10% heat-citric acid-treated soybean meal.

2) Values in the same row with different superscripts indicate significant differences between treatments (p<0.05).

SEM, standard error of the mean; BW, body weight; TDMI, total dry matter intake; CDMI, concentrate dry matter intake; FDMI, forage dry matter intake; ADG, average daily gain; FCR, feed conversion ratio (DMI/ADG); DM, dry matter; CP, crude protein; NDF, neutral detergent fiber; GE, gross energy; DE, digestible energy.

**Table 3:** Rumen fermentation characteristics in growing Hanwoo heifers fed different experimental diets

Items	Treatments^1)^		SEM	p-value^2)^

	Control	HCSBM		
NH_3_-N (mg/dL)	9.40	6.78	0.694	0.009
TVFA (mM)	73.9	70.8	2.08	0.194
Acetate (mmol/mol)	594	627	15.3	0.075
Propionate (mmol/mol)	192	172	14.3	0.197
Isobutyrate (mmol/mol)	21.6	20.5	0.831	0.244
Butyrate (mmol/mol)	134	129	3.7	0.268
Isovalerate (mmol/mol)	24.9	21.2	1.44	0.046
Valerate (mmol/mol)	33.1	30.3	3.09	0.392
A:P ratio	3.22	3.68	0.268	0.137
pH	6.58	6.64	0.060	0.319

1) Control, feed containing 10% soybean meal; HCSBM, feed containing 10% heat-citric acid-treated soybean meal.

2) Values in the same row with different superscripts indicate significant differences between treatments (p<0.05).

SEM, standard error of the mean; NH_3_-N, ammonia nitrogen; TVFA, total volatile fatty acids; A:P ratio, acetate to propionate ratio.

**Table 4:** Blood metabolites in growing Hanwoo heifers fed different experimental diets

Items	Treatments^1)^		SEM	p-value^2)^

	Control	HCSBM		
TP (g/dL)	6.59	6.44	0.152	0.363
AST (U/L)	70.3	60.4	11.48	0.426
ALT (U/L)	21.2	19.7	2.33	0.543
BUN (mg/dL)	21.8	20.5	1.31	0.387
Ca (mg/dL)	9.11	8.94	0.142	0.264
IP (mg/dL)	8.36	8.01	0.359	0.368
Mg (mg/dL)	2.41	2.29	0.058	0.077
TG (mg/dL)	12.8	12.1	1.25	0.603
Glu (mg/dL)	82.9	81.3	1.78	0.395
Alb (g/dL)	3.77	3.69	0.058	0.208
Crea (mg/dL)	0.79	0.78	0.029	0.537

1) Control, feed containing 10% soybean meal; HCSBM, feed containing 10% heat-citric acid-treated soybean meal.

2) Values in the same row with different superscripts indicate significant differences between treatments (p<0.05).

SEM, standard error of the mean; TP, total protein; AST, aspartate aminotransferase; ALT, alanine aminotransferase; BUN, blood urea nitrogen; Ca, calcium; IP, inorganic phosphorus; Mg, magnesium; TG, triglycerides; Glu, glucose; Alb, albumin; Crea, creatinine.

**Table 5:** Hematological parameters in growing Hanwoo heifers fed different experimental diets

Itemsb)	Treatments^1)^		SEM	p-value^2)^

	Control	HCSBM		
WBC (10^9^/L)	6.16	5.48	1.008	0.520
LYM (%)	68.9	62.1	5.10	0.226
MON (%)	1.67	2.46	0.502	0.162
NEU (%)	26.0	32.8	5.49	0.265
EOS (%)	4.85	2.43	1.967	0.264
BAS (%)	0.35	0.26	0.103	0.429
RBC (10^12^/L)	8.90	8.21	0.260	0.038
HGB (g/dL)	10.40	9.48	0.267	0.012
HCT (%)	34.8	31.7	0.93	0.018
PLT (10^9^/L)	96.0	91.9	31.90	0.903

1) Control, feed containing 10% soybean meal; HCSBM, feed containing 10% heat-citric acid-treated soybean meal.

b) Values in the same row with different superscripts indicate significant differences between treatments (p<0.05).

SEM, standard error of the mean; WBC, white blood cell; LYM, lymphocyte; MON, monocytes; NEU, neutrophils; EOS, eosinophils; BAS, basophils; RBC, red blood cell; HGB, hemoglobin; HCT, hematocrit; PLT, platelets.

**Table 6:** Nitrogen excretion and utilization in growing Hanwoo heifers fed different experimental diets

Items	Treatments^1)^		SEM	p-value^2)^

	Control	HCSBM		
N intake (g/d)	186	179	9.7	0.477
Urine excretion				
Creatinine (mg/dL)	50.8	35.7	7.39	0.087
Urinary N (%)	0.42	0.34	0.082	0.398
Urine output^3)^, estimated (kg/d)	4.24	6.48	1.223	0.099
Urinary N, estimated (g/d)	17.9	19.7	2.06	0.434
Fecal N (g/d)	45.9	45.7	4.18	0.966
N retention^4)^ (% of N intake)	66.0	63.4	1.49	0.139

1) Control, feed containing 10% soybean meal; HCSBM, feed containing 10% heat-citric acid-treated soybean meal.

2) Values in the same row with different superscripts indicate significant differences between treatments (p<0.05).

3) 27.11(creatinine coefficients)×BW^0.75^ ÷ [creatinine concentration(mg/dL)×10]

4) [Nitrogen intake–(Urinary nitrogen+Fecal nitrogen)/nitrogen intake]×100

SEM, standard error of the mean; BW, body weight.
